# The effects of fructose diphosphate on routine coagulation tests in vitro

**DOI:** 10.1038/s41598-021-04263-y

**Published:** 2022-01-07

**Authors:** Tongqing Chen, Duan Chen, Lu Chen, Zhengxu Chen, Baolong Wang, Daoping Zhou

**Affiliations:** 1 Blood Transfusion Department, Anhui No. 2 Provincial People’s Hospital, Hefei, Anhui China; 2 Clinical Medicine (5+3 Integration) 2020 Year 4 Class, School of Basic Medical Sciences, Wuhan University Medical School, Wuhan, Hubei China; 3 Clinical Laboratory Department, The Second People’s Hospital of Hefei, Anhui, China; 4 Clinical Laboratory Department, The First Hospital of the University of Science and Technology of China, Hefei, Anhui China; 5 Department of Oncology, Anhui No. 2 Provincial People’s Hospital, 6nd Floor,Building B, Hefei, Anhui China

**Keywords:** Diseases, Medical research, Pathogenesis, Risk factors

## Abstract

To evaluate the effects of fructose diphosphate (FDP) on routine coagulation tests in vitro, we added FDP into the mixed normal plasma to obtain the final concentration of 0, 1, 2, 3, 4, 5, 6, 10, 15, 20, 25, 30 and 35 mg/mL of drug. Prothrombin time (PT), activated partial thromboplastin time (aPTT), fibrinogen (FBG) and thrombin time (TT) of samples were analyzed with blood coagulation analyzers from four different manufacturers(Sysmex, Stago, SEKISUI and Werfen) and their corresponding reagents, respectively. Before the experiment, we also observed whether there were significant differences in coagulation test results of different lots of reagents produced by each manufacturer. At the same time as the four routine clotting tests, the Sysmex blood coagulation analyzer and its proprietary analysis software were used to detect the change of maximum platelet aggregation rate in platelet-rich plasma after adding FDP (0, 1, 2, 3, 4, 5 and 6 mg/mL). The results of PT, aPTT and TT showed a FDP (0–35 mg/mL) concentration-dependent increase and a FBG concentration-dependent decrease. The degree of change (increase or decrease) varied depending on the assay system, with PT and aPTT being more affected by the Sysmex blood coagulation testing instrument reagent system and less affected by CEKISUI, TT less affected by CEKISUI and more affected by Stago, and FBG less affected by Stago and more affected by Sysmex. The results of PT, aPTT and TT were statistically positively correlated with their FDP concentrations, while FBG was negatively correlated. The correlation coefficients between FDP and the coagulation testing systems of Sysmex, Stago, Werfen and SEKISUI were 0.975, 0.988, 0.967, 0.986 for PT, and 0.993, 0.989, 0.990 and 0.962 for aPTT, 0.994, 0.960, 0.977 and 0.982 for TT, − 0.990, − 0.983, − 0.989 and − 0.954 for FBG, respectively. Different concentrations of FDP (0, 1, 2, 3, 4, 5 and 6 mg/mL) had different effects on the maximum aggregation rate of platelet induced by the agonists of adenosine diphosphate (ADP, 5 µmol/L), arachidonic acid (Ara, 1 mmol/L), collagen (Col, 2.5 µg/mL) and epinephrine (Epi,10 µmol/L), but the overall downward trend was consistent, that is, with the increase of FDP concentration, the platelet aggregation rate decreased significantly. Our experimental study demonstrated a possible effect of FDP on the assays of coagulation and Platelet aggregation, which may arise because the drug interferes with the coagulation and platelet aggregation detection system, or it may affect our in vivo coagulation system and Platelet aggregation function, the real mechanism of which remains to be further verified and studied.

## Introduction

The primary and basic energy for cellular activity in human tissues is derived from the aerobic oxidation of glucose and/or anaerobic enzymes to produce adenosine triphosphate (ATP)^1^. Fructose diphosphate (FDP), also known as fructose 1,6-diphosphate (FDP), is an important intermediate metabolite produced during glycolysis in cellular energy metabolism and is an effective mediator of glycolysis, promoting glycolysis, improving glycolytic efficiency, regulating intracellular K^+^ and Ca^2+^ concentrations, inhibiting intracellular oxygen radical production, and maintaining cell membrane stability^2,3^. Clinical studies have shown that exogenous FDP, when injected into humans, exerts protective and anti-damaging effects in a variety of cells and tissues, including brain, kidney, intestine, liver, and heart, and also reduces morbidity and mortality in patients with sepsis^4,5^. It has also been found that exogenous FDPs have few toxic side effects during administration, few contraindications, can be administered orally or intravenously, and are widely used in the clinical setting^2,3,5,6^.

Routine coagulation tests, including prothrombin time (PT), activated partial thromboplastin time (aPTT), fibrinogen (FBG) and thrombin time (TT), are one of the most common tests performed in clinical laboratory departments, and are mandatory before surgery, as well as for monitoring thrombophilia and oral anticoagulants. However, the test results are often affected or interfered by many factors, such as drugs, telavancin (Vibativ)^7^, oritavancin^8^, pentasaccharide^9^, etc. FDP is a cytoprotective agent that is widely used in the treatment of cellular injury diseases of heart, brain, liver, kidney and lung tissues. There are only sporadic reports on the side effects of this drug, and little is known so far about whether this drug has an effect on blood coagulation. In this paper, we use in vitro experiments to investigate whether FDP has an effect on coagulation results.

## Results

### Coagulation test results for different lots of reagents from each manufacturer (Sysmex, Stago, SEKISUI and Werfen)

The 21 random outpatient or inpatient samples results of PT, aPTT, FBG and TT tested by the Sysmex, Stago, SEKISUI and Werfen coagulation test system with two different lots of reagents provided by the manufacturer were showed in Table 1 and cont. There were no statistically significant differences between the two lots of reagents from each manufacturer (*P* > 0.05).

**Table 1:** The results of plasma PT (s), aPTT (s), FIB (g/L) and TT (s) were determined by Sysmex, Stago, Werfen and CEKISUI coagulation detection system with two different lots reagents($\overline{x}$ ± *s*, n = 21).

Sample	PT (s) (Sysmex)		APTT (s) (Sysmex)		FIB (g/L) (Sysmex)		TT (s) (Sysmex)	
	Reagent lot: 565738	Reagent lot: 565756	Reagent lot: 557639	Reagent lot: 557641	Reagent lot: 563527	Reagent lot: 563530	Reagent lot: 562080	Reagent lot: 565094
21 samples	12.4 ± 1.13	12.3 ± 1.22	30.6 ± 2.95	30.9 ± 2.67	3.4 ± 0.54	3.40 ± 0.52	17 ± 1.47	17.2 ± 1.55
*t*	1.08		1.12		0.14		1.46	
*P*	0.29		0.28		0.89		0.16	

Sample	PT (s) (Stago)		APTT (s) (Stago)		FIB (g/L) (Stago)		TT (s) (Stago)	
	Reagent lot: 257594	Reagent lot: 257684	Reagent lot: 257254	Reagent lot: 258900	Reagent lot: 257491	Reagent lot: 257900	Reagent lot: 256890	Reagent lot: 258820
21 samples	12.3 ± 1.33	12.3 ± 1.28	36.1 ± 5.74	35.8 ± 5.78	3.70 ± 0.41	3.7 ± 0.36	18.1 ± 2.22	18 ± 2.26
*t*	0.31		0.91		0.11		0.16	
*P*	0.76		0.37		0.92		0.88	

Sample	PT (s) (Werfen)		APTT (s) (Werfen)		FIB (g/L) (Werfen)		TT (s) (Werfen)	
	Reagent lot: N0797389	Reagent lot: N1199931	Reagent lot: N0897668	Reagent lot: N0101208	Reagent lot: N1190250	Reagent lot: N0714362	Reagent lot: N0998913	Reagent lot: N0202256
21 samples	12.0 ± 0.99	12.0 ± 0.85	33.6 ± 3.9	33.7 ± 3.5	3.60 ± 0.40	3.60 ± 0.30	12.5 ± 1.61	12.3 ± 1.72
*t*	0.07		1.45		0.96		1.22	
*P*	0.95		0.16		0.35		0.24	

Sample	PT (s) (CEKISUI)		APTT (s) (CEKISUI)		FIB (g/L) (CEKISUI)		TT (s) (CEKISUI)	
	Reagent lot: 826RBS	Reagent lot: 847RIQ	Reagent lot: 826RJQ	Reagent lot: 834RBS	Reagent lot: 801RKQ	Reagent lot: 817RGS	Reagent lot: SF001	Reagent lot: SF003
21 samples	12.2 ± 1.06	12.1 ± 0.98	31.3 ± 2.85	31.4 ± 2.72	3.50 ± 0.53	3.50 ± 0.53	16.2 ± 2.07	16.3 ± 1.96
*t*	1.08		0.46		1.11		1.41	
*P*	0.29		0.65		0.28		0.17	

*PT* The prothrombin time, *aPTT* activated partial thromboplastin time, *FBG* fibrinogen, *TT* thrombin time, *Sysmex* Sysmex blood coagulation testing instrument reagent system, *Stago* Stago blood coagulation testing instrument reagent system, *Werfen* Werfen blood coagulation testing instrument reagent system, *CEKISUI* CEKISUI blood coagulation testing instrument reagent system.

### The relationship between plasma FDP concentration and coagulation results

After the addition of FDP (0–35 mg/mL) to mixed plasma in vitro, PT, aPTT and TT assays showed a concentration-dependent prolongation and a concentration-dependent decrease of FBG, with the degree of prolongation or decrease varying depending on the assay system; when the plasma FDP concentration was 35 mg/mL, the aPTT assay results detected by the Stago and Werfen Analysis system were shown to be outside the detection range of the instrument and did not display the results correctly (Table 2).

**Table 2:** PT (s), aPTT (s), FIB (g/L) and TT (s) of the pooled plasma containing with different concentrations FDP in vitro detected by Sysmex, Stago, Werfen, and CEKISUI blood coagulation analysis system($\overline{x}$ ± *s*, *n* = 3).

FDP(mg/mL)	PT(s)				aPTT(s)			
	Sysmex	Stago	Werfen	CEKISUI	Sysmex	Stago	Werfen	CEKISUI
0	12.4 ± 0.23	12.8 ± 0.17	11.7 ± 0.23	12.9 ± 0.15	26.9 ± 0.32	41.3 ± 0.44	35.8 ± 0.29	36.7 ± 0.62
1	12.6 ± 0.17	12.9 ± 0.25	11.9 ± 0.25	13.1 ± 0.17	28.7 ± 0.35	44.4 ± 0.56	38.4 ± 0.26	37.9 ± 0.44
2	12.8 ± 0.15	13.3 ± 0.21	11.7 ± 0.32	13.0 ± 0.21	29.8 ± 0.25	46.4 ± 0.42	39.9 ± 0.64	39.6 ± 0.15
3	12.9 ± 0.35	13.5 ± 0.15	11.9 ± 0.25	13.2 ± 0.29	35.0 ± 0.38	50.1 ± 0.55	45.5 ± 0.32	41.3 ± 0.15
4	13.6 ± 0.15	13.5 ± 0.21	12.2 ± 0.15	13.6 ± 0.12	36.9 ± 0.32	54.6 ± 0.50	48.5 ± 0.53	42.4 ± 0.23
5	14.2 ± 0.25	14.1 ± 0.15	12.4 ± 0.46	14.2 ± 0.25	41.4 ± 0.21	58.4 ± 0.56	55.6 ± 0.21	44.0 ± 0.64
6	14.8 ± 0.21	14.9 ± 0.18	12.7 ± 0.17	14.3 ± 0.26	45.4 ± 0.23	60.7 ± 0.59	59.8 ± 0.35	44.4 ± 0.78
7	14.8 ± 0.17	15.2 ± 0.26	12.8 ± 0.35	14.6 ± 0.01	51.4 ± 0.15	64.7 ± 0.32	65.3 ± 0.72	48.1 ± 0.92
10	16.6 ± 0.25	15.8 ± 0.45	13.4 ± 0.25	15.0 ± 0.17	59.4 ± 0.40	72.0 ± 0.95	70.2 ± 1.88	54.5 ± 0.59
15	19.2 ± 0.70	18.2 ± 0.26	15.6 ± 0.20	16.3 ± 0.15	74.5 ± 1.91	91.1 ± 1.14	91.7 ± 1.15	57.9 ± 1.39
20	24.0 ± 0.23	20.7 ± 0.53	16.6 ± 0.44	19.5 ± 0.44	101.5 ± 2.15	125.3 ± 3.06	132.6 ± 3.09	77.0 ± 0.85
25	29.1 ± 0.59	23.5 ± 0.38	20.2 ± 0.36	21.3 ± 0.12	129.1 ± 4.61	165.6 ± 4.05	174.4 ± 3.51	134.4 ± 1.97
30	34.6 ± 0.45	25.8 ± 0.51	22.2 ± 0.38	23.1 ± 0.25	150.5 ± 6.71	178.0 ± 6.00	190.4 ± 3.81	139.8 ± 2.52
35	44.4 ± 0.81	31.1 ± 0.84	28.0 ± 0.52	27.0 ± 0.53	189.5 ± 8.67			145.8 ± 2.28
*r*	0.975	0.988	0.967	0.986	0.993	0.989	0.990	0.962
*t*_*r*_	15.105	22.478	13.218	20.718	28.461	21.827	23.102	12.240
Regression equation	*Y*_*result*_ = 0.836*X*_*FDP*_ + 9.986	*Y*_*result*_ = 0.489*X*_*FDP*_ + 11.831	*Y*_*result*_ = 0.416*X*_*FDP*_ + 10.387	*Y*_*result*_ = 0.382*X*_*FDP*_ + 12.056	*Y*_*result*_ = 4.457*X*_*FDP*_ + 19.536	*Y*_*result*_ = 4.712*X*_*FDP*_ + 34.571	*Y*_*result*_ = 5.333*X*_*FDP*_ + 28.116	*Y*_*result*_ = 3.410*X*_*FDP*_ + 27.712
*P*	< 0.000	< 0.000	< 0.000	< 0.000	< 0.000	< 0.000	< 0.000	< 0.000

FDP (mg/mL)	FBG (g/L)				TT (s)			
	Sysmex	Stago	Werfen	CEKISUI	Sysmex	Stago	Werfen	CEKISUI
0	3.59 ± 0.16	3.53 ± 0.12	3.31 ± 0.13	3.58 ± 0.06	17.9 ± 0.49	18.2 ± 0.64	12.2 ± 0.12	17.3 ± 0.15
1	3.51 ± 0.14	3.54 ± 0.13	3.29 ± 0.15	3.53 ± 0.10	18.4 ± 0.31	19.4 ± 0.32	12.3 ± 0.38	17.4 ± 0.20
2	3.49 ± 0.11	3.51 ± 0.16	3.32 ± 0.09	3.51 ± 0.07	19.1 ± 0.61	20.3 ± 0.46	12.9 ± 0.25	17.4 ± 0.35
3	3.36 ± 0.13	3.45 ± 0.13	3.34 ± 0.13	3.39 ± 0.07	20.3 ± 0.72	22.6 ± 0.31	13.5 ± 0.35	18.4 ± 0.25
4	3.30 ± 0.10	3.42 ± 0.16	3.29 ± 0.13	3.30 ± 0.08	20.4 ± 0.67	23.5 ± 0.67	14.4 ± 0.26	19.2 ± 0.35
5	3.14 ± 0.12	3.43 ± 0.20	3.26 ± 0.10	3.15 ± 0.10	21.0 ± 0.84	25.0 ± 0.85	14.7 ± 0.32	19.6 ± 0.30
6	3.12 ± 0.14	3.37 ± 0.13	3.26 ± 0.17	3.09 ± 0.08	22.1 ± 0.57	26.4 ± 0.61	15.4 ± 0.47	20.5 ± 0.21
7	3.11 ± 0.15	3.38 ± 0.12	3.19 ± 0.17	2.97 ± 0.09	23.0 ± 0.51	28.4 ± 0.49	16.3 ± 0.23	20.9 ± 0.60
10	2.97 ± 0.17	3.35 ± 0.14	3.12 ± 0.12	2.93 ± 0.08	26.8 ± 0.89	31.0 ± 0.81	17.7 ± 0.53	22.4 ± 0.38
15	2.82 ± 0.13	3.30 ± 0.16	3.04 ± 0.16	2.86 ± 0.08	30.8 ± 1.15	38.8 ± 1.48	20.5 ± 0.67	25.8 ± 0.35
20	2.51 ± 0.14	3.08 ± 0.12	2.77 ± 0.12	2.69 ± 0.12	38.6 ± 1.01	52.7 ± 1.42	28.1 ± 0.35	30.9 ± 0.90
25	2.30 ± 0.11	3.05 ± 0.10	2.66 ± 0.14	2.58 ± 0.10	45.6 ± 1.41	71.5 ± 2.17	34.2 ± 0.67	38.1 ± 0.50
30	2.19 ± 0.11	3.00 ± 0.14	2.46 ± 0.11	2.51 ± 0.12	50.0 ± 1.78	77.0 ± 1.54	38.0 ± 1.37	39.6 ± 1.00
35	1.96 ± 0.11	2.95 ± 0.16	2.42 ± 0.13	2.31 ± 0.07	60.0 ± 1.91	121.3 ± 4.55	51.5 ± 1.35	51.1 ± 1.30
*r*	− 0.990	− 0.983	− 0.989	− 0.954	0.994	0.960	0.977	0.982
*t*_*r*_	− 24.455	− 18.632	− 23.695	− 11.029	31.384	11.888	15.932	18.114
Regression equation	*Y*_*result*_ = − 0.045 *X*_*FDP*_ + 3.482	*Y*_*result*_ = − 0.017*X*_*FDP*_ + 3.515	*Y*_*result*_ = − 0.029*X*_*FDP*_ + 3.384	*Y*_*result*_ = − 0.034*X*_*FDP*_ + 3.422	*Y*_*result*_ = 1.176*X*_*FDP*_ + 15.879	*Y*_*result*_ = 2.496*X*_*FDP*_ + 12.094	*Y*_*result*_ = 1.018*X*_*FDP*_ + 9.701	*Y*_*result*_ = 0.894*X*_*FDP*_ + 15.211
*P*	< 0.000	< 0.000	< 0.000	< 0.000	< 0.000	< 0.000	< 0.000	< 0.000

*FDP* Fructose diphosphate, *PT* The prothrombin time, *aPTT* activated partial thromboplastin time, *FBG* fibrinogen, *TT* thrombin time, *Sysmex* Sysmex blood coagulation testing instrument reagent system, *Stago* Stago blood coagulation testing instrument reagent system, *Werfen* Werfen blood coagulation testing instrument reagent system, *CEKISUI* CEKISUI blood coagulation testing instrument reagent system.

**Figure 1:**
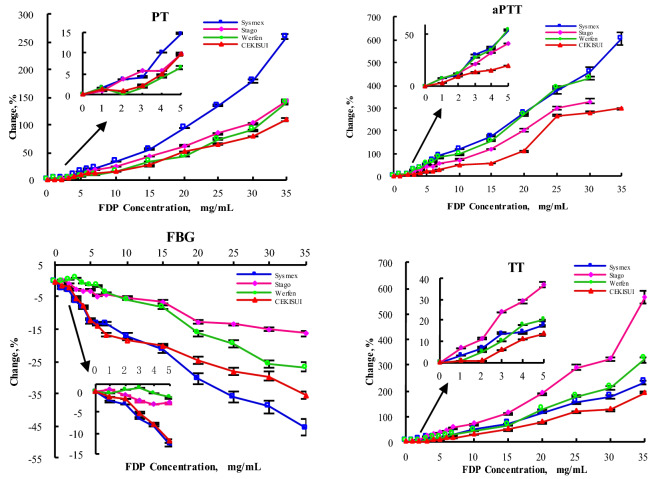
Percentage change in PT (s), aPTT (s), FBG (g/L) and TT (s) of the pooled normal plasma after adding with different concentrations FDP in vitro detected respectively by Sysmex, Stago, Werfen, and CEKISUI blood coagulation detection system. The results of PT, aPTT and TT were increased in a FDP concentration (0–35 mg/mL) dependent way after the addition of FDP in the mixed plasma in vitro, while FBG was decreased in a concentration dependent way. When the concentration of FDP was 35 mg/mL, APTT detected by Stago and Werfen blood coagulation system, the analysis system indicated that it was out of the detection range of the instrument and could not display the results correctly. The degree of extension or decrease varied with different detection systems. When the concentration of FDP was 0–35 mg/mL, PT was significantly affected by the Sysmex detection system (0–258.8%), but was less affected by SEKISUI (0–108.8%). FBG was less affected by Stago (− 16.3–0%) and more affected by Sysmex (− 45.1–0%).TT was more influenced by Stago (0–565.4%) and less by SEKISUI (0–194.8%). When the concentration of FDP was 0–30 mg/mL, APTT was significantly affected by the Sysmex detection system (0–439.9%), but was less affected by SEKISUI (0–280.9%). *FDP* Fructose diphosphate, *PT* prothrombin time, *aPTT* activated partial thrombin time, *TT* thrombin time, *FBG* fibrinogen.

Compared to the four coagulation assay systems, the variation of plasma FDP concentration on PT and aPTT results was more pronounced in the Sysmex assay system and less influenced by SEKISUI, TT less affected by CEKISUI and more affected by Stago, and FBG was less influenced by the Stago assay system and more influenced by Sysmex. When plasma FDP concentrations ranged from 5 to 10 mg/mL, the FBG assay showed insignificant changes in the Werfen assay system (− 1.51% to − 5.74%), while the Stago assay system showed insignificant changes in FBG assay results when plasma FDP concentrations ranged from 0 to 15 mg/mL (− 6.52%–0%). The change of plasma FDP effect on the results of PT, aPTT, TT and FBG coagulation assays in four different coagulation testing systems is shown in Fig. 1.

### Correlation between plasma coagulation results and FDP concentration

Statistical analysis software SPSS22.0 correlation analysis showed that plasma PT, aPTT and TT test results were positively correlated with their FDP concentrations, while FBG was negatively correlated; the correlation coefficients varied according to the different coagulation testing systems. The correlation coefficients between FDP and the coagulation testing systems of Sysmex, Stago, Werfen and SEKISUI were 0.975, 0.988, 0.967, 0.986 for PT, and 0.993, 0.989, 0.990 and 0.962 for aPTT, 0.994, 0.960, 0.977 and 0.982 for TT, − 0.990, − 0.983, − 0.989 and − 0.954 for FBG, respectively. Statistical hypothesis tests were performed on the correlation coefficients, and the results showed that the correlation coefficients between the plasma result of FBG, PT, aPTT and TT respectively tested by the four coagulation assay systems and plasma FDP concentrations of were extremely statistically significant (*P* < 0.000), and the results are shown on Table 2.

### Platelet aggregation test results

**Figure 2:**
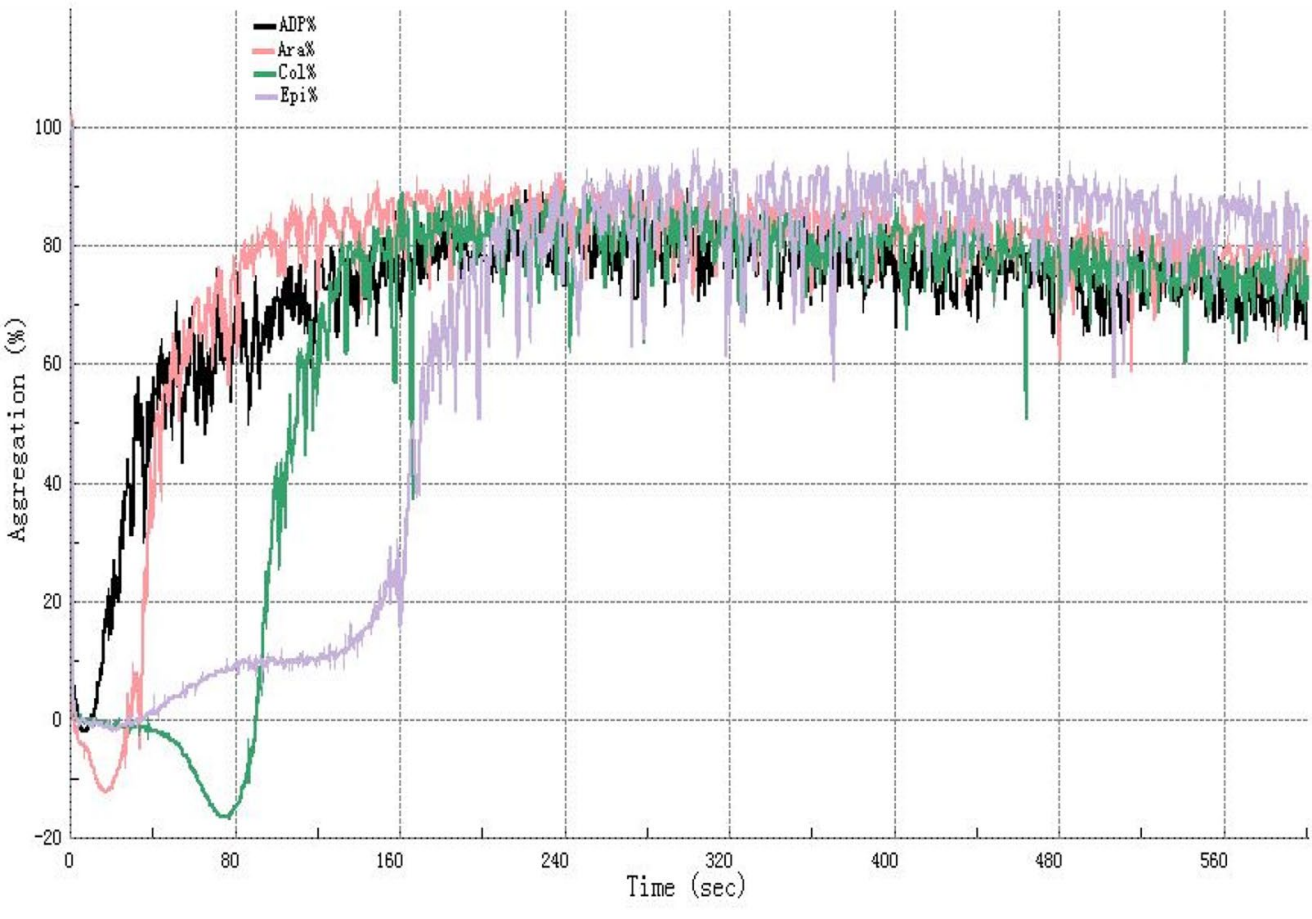
Representative platelet aggregation rate (Aggregation%) curve of normal healthy volunteers after adding ADP (5 µmol/L), Ara (1 mmol/L), Col (2.5 µg/mL) or Epi(10 µmol/L) into platelet-rich plasma, respectively. It can be seen from the figure that the platelet time-dependent aggregation response curves caused by different platelet stimulants are not completely consistent. *ADP* Adenosine diphosphate, *Ara* arachidonic acid, *Epi* epinephrine, *Col* collagen.

**Figure 3:**
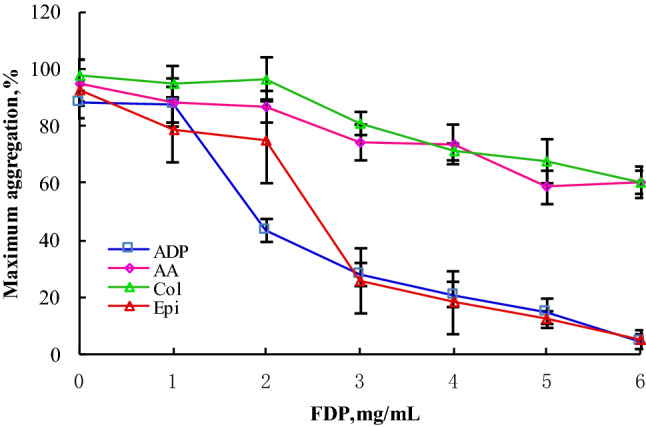
The maximum aggregation rate (MA%) of platelets in platelet-rich plasma from 5 healthy adults added with different concentrations of FDP in vitro was determined by the Sysmex CS5100 coagulation analyzer system ($\overline{x}$ ± s, n = 5). With the increase of FDP concentration (0, 1, 2, 3, 4, 5 and 6 mg/mL), The maximum value of platelet aggregation activated by ADP (5 µmol/L), Ara (1 mmol/L), Col (2.5 µg/mL) and Epi (10 µmol/L) showed a significant downward trend. When the plasma FDP concentration increased from 1 to 2 mg/mL, the maximum aggregation rate of platelet aggregation induced by ADP agonist decreased significantly from (87.3 ± 6.1)% (n = 5) to (43.5 ± 4.3)% (n = 5). When the plasma FDP concentration increased from 2 to 3 mg/mL, the maximum aggregation rate of platelet aggregation induced by Epi agonist decreased significantly from (74.8 ± 14.5)% (n = 5) to (25.9 ± 11.5%) (n = 5). *ADP* Adenosine diphosphate, *Ara* arachidonic acid, *Epi* epinephrine, *Col* collagen.

The maximum aggregation rate (MA%) of 5 samples ($\overline{x}$ ± SD, n = 5) and representative aggregation with time-dependent reaction curve of platelet-rich plasma containing FDP drugs at different concentrations under the agonists of adenosine diphosphate (ADP, 5 µmol/L), arachidonic acid (Ara, 1 mmol/L), collagen (Col, 2.5 µg/mL) and epinephrine (Epi, 10 µmol/L) are shown in Figs. 2 and 3. The effects of different concentrations of FDP on the maximum platelet aggregation rate induced by the four agonists were different, but the overall downward trend was consistent, that is, with the increase of FDP concentration, the platelet aggregation rate decreased significantly.

## Discussion

Our experiments in vitro revealed a significant effect of FDP on coagulation test results. The National Drug and Food Administration recommends a dosage of FDP of 5–10 g per day, according to which we calculated the plasma concentration of FDP after intravenous administration to be about 3.18–6.35 g/L, based on the conversion of body weight and plasma volume in adults, assuming a body weight of 50 kg and a theoretical extrapolation of plasma volume of about 1.575 L. In this study, we observed that plasma PT, APTT and TT were prolonged when 5 mg/mL FDP was added in plasma, and the experimental results also showed that plasma PT, APTT and TT were significantly prolonged with the increase of drug concentration in a dose-dependent.

PT, aPTT and TT reflect the function of extrinsic, intrinsic and common coagulation pathways. This assay in vitro is performed by adding different reagent manufacturers producing some form of thrombin, tissue factor or other components such as phospholipids, surface activators, calcium ions, etc. It has been reported in the literature that FDP resists platelet aggregation by inhibiting a variety of platelet aggregation inducers, including ADP. The role of platelet procoagulation mainly involves platelet factor 3 (PF3) in platelet phospholipids, which is exposed to the platelet coat after platelet aggregation activation and completes the activation of coagulation factor X and prothrombin on the surface of PF3^10^. We therefore hypothesized that the possible inhibition of platelet aggregation by FDP would affect the conversion of prothrombin to thrombin, thereby prolonging PT, APTT and TT. The results of this study showed that FDP (0–6 mg/mL) was concentration-dependent on platelet aggregation induced by AA, ADP, Coland Epi. AA produces intracyclic peroxide under the action of intracyclic peroxidase, synthesizes thromboxane A2 (TXA2) under the action of thromboxane synthase, and then binds to TXA2 receptor of platelets to activate platelets and aggregate. ADP acts on platelet GP vi and α2β2, Col acts on platelet GP vi and α2β2, AND ADP acts on P2Y1 and P2Y12 receptors, thus introducing ADP to accelerate platelet aggregation^11^. It is generally believed that phosphorylated sugars do not penetrate cell membranes^11^, but some scholars believe that FDP can enter the cell and phosphorylate 47 kDa protein in the cell, thus inhibiting platelet aggregation-induced by platelet inducer^12^. The author thinks that, as a result of the four platelet activator activation have been varying degrees of inhibition, FDP, as a kind of small molecules, it is hard to imagine through four mechanisms restrain excited agent platelet aggregation, so we agree with the latter point of view, namely the FDP into intracellular 47 kDa protein phosphorylation, thereby inhibition of platelet aggregation. Cyclic nucleotide CAMP and cGMP are effective inhibitors of platelet activation^13^, and it has been reported that there is no significant change in intracellular CAMP level after FDP treatment. Therefore, it can be ruled out that FDP leads to the increase of intracellular CAMP and cGMP in platelet and causes the decrease of platelet aggregation function^11^. The mechanism of FDP leading to reduced platelet aggregation remains to be further studied.

The degree of PT and APTT and TT prolongation also differed on the different reagent instrumentation assay systems, with a significantly higher degree of variation between the assay systems of the Sysmex hemagglutinator reagent than the other three assay systems at the same drug concentration. According to previous reports, this difference may be closely related to the composition of the reagents and the source of the phospholipids. Further comparative studies could not be done because the composition of reagents from different manufacturers is a matter of commercial confidentiality^14^. As the specific details of commercially determined chemical composition are proprietary and known only to the manufacturer^14^, it is not possible to determine the basis for differences in test results obtained using different reagents. Coagulation test results are often affected by a variety of factors, such as testing equipment, testing principle and methodology, reagent composition and content, as well as testing conditions and procedural parameters, especially the different sources of reagent raw materials, etc. Even if the same sample is provided with detection systems by different manufacturers, the results are often very different. For example, TT results of the same sample measured by Stago coagulation apparatus exceed Werfen detection by nearly 50%. Therefore, the reference range of normal people suggested by manufacturers of different detection systems is also different. For example, the TT reference range of Stago kit instruction is 14–21 s, while Werfen is 10.3–16.6 s. Therefore, it is of little significance to simply analyze and compare the differences in the detection results of different detection systems. In this study, four coagulation detection systems from different manufacturers were used to analyze the influence of FDP on coagulation results. The main purpose of this study is to verify the influence of FDP on coagulation results through more coagulation detection systems. To prevent false anomalies caused by accidental factors of a single manufacturer or technical causes of a single system, the purpose of our study is not to compare the differences of coagulation test results of different manufacturers, but to show that the influence of FDP on coagulation test results has been fully verified in multiple detection systems. Given that the reagents of the Sysmex instrument reagent assay system exhibit a higher sensitivity to the FDP drug, then more care should be taken to avoid the use of this family of reagents when performing coagulation tests on patients who have used this drug. On the other hand, if the clotting system in the body is really affected, it is necessary to ask clinicians to closely observe the occurrence of abnormal clotting in patients using the drug.

In this experiment, FBG assays were performed on 14 samples with graded FDP concentrations using four instrumental reagent assay systems, and the results showed a concentration-dependent decrease in FDP assay results using the Sysmex, Werfen, and SEKISUI assay systems. In contrast, the changes in fibrinogen were not significant with increasing drug concentrations when tested on the stago instrumental reagent assay system. The principle of interference of FDP on fibrinogen determination is not clear and may be related to thrombin production.

FDP is a biochemically active substance in the glucose metabolism and energy metabolism of human tissue cells, which can effectively provide bioenergy, promote the metabolic activities of biological tissue cells, and enhance the functional activities of various tissues and organs. It is conducive to repairing cell damage. It improves cardiac contractility, increases cardiac blood transfusion, and maintains better hemodynamics under ischemic and hypoxic conditions. Therefore it has a wide range of uses in the treatment of cardiovascular diseases. During the treatment of cardiovascular diseases, antithrombotic drugs are often used, which may affect the coagulation function. Therefore most patients undergoing cardiovascular therapy are subjected to in vitro coagulation monitoring^5^. Although this trial revealed the effect of FDP on in vitro coagulation assays, clinical care is needed to exclude the effect of FDP on coagulation assays when used in combination and to collect coagulation specimens prior to the use of FDP. We have not performed in vivo trials and it is unclear whether the drug causes bleeding risk, but clinicians should be concerned about whether there is an effect on coagulation and should also carefully assess coagulation and bleeding tendency when using the drug.

Our study has some limitations. We investigate whether in vitro FDP has an effect on coagulation outcomes by hypothesizing that the initial experimental drug concentrations were obtained based on the presumed doses used in the drug's instructions for use, with expected plasma concentrations of 2.25–7.75 mg/mL and theoretical presumed values of about 5 mg/mL. A series of experimental concentrations were artificially set up to 35 mg/mL, with the aim of verifying that in vitro FDP The purpose of the series of experiments was to verify the correlation between in vitro FDP concentration and coagulation results, not the in vivo administration concentration, and its true in vivo concentration, which remains to be verified by pharmacokinetic or pharmacodynamic data of the drug. In-vitro potentially useful study is useful to display potential agents between reagent platforms, But first, we are curious to know if there is any indication that when the fructose diphosphate is given, there are alterations in treated patients. We look forward to the next study that may be useful is the collection of blood samples at baseline and after fructose diphosphate infusion, following peak measurements for pharmacokinetic (PK) studies.

In summary, our studies in vitro confirm that FDP affects our coagulation assay results and platelet aggregation, and whether FDP actually affects our human coagulation function and platelet aggregation should attract our extensive attention. We would like to speculate that some pharmacological effects of FDP, such as improvement of ischemic and hypoxic tissue injury during reperfusion^15^, may be related to the inhibition of platelet activation and anticoagulation described in this paper. If possible, we will soon conduct in vivo experimental studies and look forward to achieving the desired results.

## Methods

### Instruments and reagents

PT, aPTT, FBG and TT of samples were analyzed with blood coagulation analyzers from four different manufacturers (Sysmex, Stago, SEKISUI and Werfen) and their corresponding reagents, respectively. The Sysmex blood coagulation instrument reagent testing system is based on the Sysmex CS5100 blood coagulation analyzer and its corresponding reagents with Dade Actin Activated Cephaloplastin Reagent for aPTT test, Thromborel S for PT, Dade Thrombin Reagent for FBG, and Test Thrombin Reagent for TT (all from Siemens Healthcare GmbH, Erlangen, Germany). The Stago blood coagulation instrument reagent testing system used the STA-R Evolution coagulation analyzer and the reagents of STA-PTT for aPTT, STA-Neoplastine CI Plus for PT, STA-Fibrinogen for FBG and STA-Thrombin for TT (all from Stago Diagnostica, Diagnostica Stago S.A.S, Asnières sur Seine, France). The SEKISUI blood coagulation instrument reagent testing system: Analysis with SEKISUI CP2000 coagulation system, Reagent of Coagpia PT-N for PT, Coagpia aPTT-N for aPTT, Thrombin time test kit for TT, and Coagpia FBG for FBG were obtained from SEKISUI (SEKISUI MEDICAL CO., LTD. Tokyo, Japan.). The Werfen blood coagulation instrument reagent testing system: HemosIL RecombiPlasTin 2G for PT, HemosIL SynthAsil for aPTT, HemosIL Thrombin Time for TT, and HemosIL Fibrinogen-C XL for FBG (Instrumentation Laboratory, Milan, Italy) were purchased and used with the ACL-TOP 500 coagulation system. FDP was purchased from Centrin Pharmaceutical Co., Ltd, Jining, Shandong, China. The agonists of adenosine diphosphate (ADP, 5 µmol/L), arachidonic acid (Ara 1 mmol/L), collagen (Col, 2.5 µg/mL) and epinephrine (Epi, 10 µmol/L) with prototype software were manufactured and supplied by the Sysmex company, Japan.

### Sample collection and methods

The following experimental procedures and protocols were approved by the ethical review committee of Anhui No.2 Provincial people’s Hospital [No. (R)2021-008]. Fresh specimens of 3.2% trisodium citrate anticoagulation (blood:citric acid, 9:1) were randomly obtained from 6 voluntary and healthy adults with normal results of the four routine coagulation tests (no history of blood disorders such as platelets and coagulation dysfunction, and no medications that have an effect on coagulation such as aspirin within the last 2 weeks). Among the 6 participants, 5 with platelet counts greater than 150 × 10^9^/L but less than 450 × 10^9^/L were selected for platelet aggregation experiment. The specimens used for coagulation tests were centrifuged and the upper layer of plasma was taken, collected and mixed thoroughly to prepare a mixed fresh. The plasma was sealed and packed in – 70 °C refrigerator for freezing and storage, and set aside. The samples were thawed in a 37 °C water bath for 5 min before the experiment, gently shaken and mixed, dissolve FDP into a series of concentration solutions according to the special solvent provided in the FDP instructions and 10 μL FDP with different concentrations was added to the 2 mL mixed plasma for obtaining mixed drug plasma with final concentrations of 0 (add 10 μL FDP solution), 1, 2, 3, 4, 5, 6, 7, 10, 15, 20, 25, 30, and 35 mg/mL (the dose of human FDP approved by the State Food and Drug Administration is 5–10 g per day, which corresponds to a human plasma concentration of 2.25–7.75 mg/mL, approximately. With average concentration nearly 5 mg/mL, so the design starting concentration of this experiment is 1 mg/mL). The assay was performed according to the manufacturer's procedures, and the results of PT, aPTT, FBG and TT were measured for each specimen in the four coagulation assay systems described above (Sysmex, Stago, SEKISUI and Werfen), all of which were coagulation methods, but the SEKISUI coagulation assay systems PT and aPTT were coagulation time methods and FBG was thrombin time method. Dissolve the FDP with the matching solution according to the drug instructions. Before this test, in addition to the normal calibration, all assay systems were conducted with commercially available control samples, specified by the manufacturers of each assay system supporting traceability, including normal and abnormal quality control samples. With the assay system in normal condition, each sample was then tested on the machine.

### Comparison of coagulation test results with different lots of reagents produced by each manufacturer (Sysmex, Stago, SEKISUI and Werfen)

Using two different lots of reagents provided by the manufacturer with each coagulation test system (Sysmex, Stago, SEKISUI and Werfen), a total of 21 random outpatient or inpatient samples were tested for four coagulation tests (PT, aPTT, FBG and TT).

### Platelet aggregation tests

We used the Sysmex CS5100 coagulation analyzer and the agonists ADP (5 µmol/L), Ara (1 mmol/L), Col (2.5 µg/mL) and Epi (10 µmol/L)^16^ with its proprietary analysis software to perform aggregation in platelet-rich plasma (PRP) derived from 5 normal healthy participants, whose platelet count is 150 ~ 450 × 10^9^/L. The platelet-rich plasma was prepared according to the instructions provided by the Sysmex manufacturer, and FDP was added so that the final concentration of the plasma was 0, 1, 2, 3, 4, 5 and 6 mg/mL, respectively. The experiments were completed in four hours. The platelet maximum aggregation rate (MA%) was obtained by its analysis software.

### Statistical analysis

Each sample was tested in three independent replicates according to the manufacturer's protocol and completed within two hours, and the average of the three assay results was used as the basis for calculation. The coagulation test results of samples with FDP concentration of 0 mg/mL were used as control tubes, and the percentage change of each sample test result from the control tube was calculated to infer whether these drugs had an effect on the coagulation test results based on the degree of change, and to further assess whether there was concentration-dependent interference in the coagulation test. All statistical analyses were performed using Microsoft Excel 2003 (Microsoft Corporation, Redmond, WA, USA), using Statistical Product and Service Solutions 20.0 (SPSS20.0, IBM, Armonk, USA) statistical software linear regression analysis the coefficients of correlation between PT, aPTT, FIB, or TT measurements of specimens and FDP concentrations for each assay system to obtain linear regression equations, respectively, and statistical hypothesis tests were performed on the regression coefficients to determine whether a linear relationship existed, and *P* < 0.05 was used to determine whether there was significant significance. The influence of different lots of reagents on the results of the same coagulation test was statistically analyzed by paired T-test.

### Human and animal rights

All procedures performed in study involving human participants were in accordance with the ethical standards of the institutional and/or national research committee and with the 1964 Helsinki declaration and its later amendments or comparable ethical standards. The study was approved by our Institutional Review Board.

### Ethical approval

The purpose of this study was to investigate the effects of FDP on routine coagulation tests in vitro. This study was conducted in vitro and has no adverse effects on patients’ health, because the samples used were the remaining samples after clinical examination and experimenters did not have direct contact with patients. Demographic and clinical data were collected by a questionnaire. The the Ethical Committees of The Anhui No.2 Provincial People's Hospital approved the study [protocol number: No. (R)2021-008] and all the participants provided their written informed consent in accordance with the Declaration of Helsinki.

## References

[1] Zhang CS (2017). Fructose-1,6-bisphosphate and aldolase mediate glucose sensing by AMPK. Nature.

[2] Shams F, Oldfield NJ, Wooldridge KG, Turner DPJ (2014). Fructose-1,6-bisphosphate aldolase(FBA)-a conserved glycolytic enzyme with virulence functions in bacteria: 'ILL met by moonlight'. Biochem. Soc. Trans..

[3] Murakami K, Yoshino M (2015). Effect of fructose 1,6-bisphosphate on the iron redox state relating to the generation of reactive oxygen species. Biometals.

[4] Wang W, Liu M, You C, Li Z, Zhang YHP (2017). ATP-free biosynthesis of a high energy phosphate metabolite fructose 1,6-diphosphate by in vitro metabolic engineering. Metab. Eng..

[5] Alva N, Alva R, Carbonell T (2016). Fructose 1,6-bisphosphate: A summary of its cytoprotective mechanism. Curr. Med. Chem..

[6] Li TT, Xie JZ, Wang L, Gao YY, Jiang XH (2015). Rational application of fructose-1, 6-diphosphate: From the perspective of pharmacokinetics. Acta Pharm..

[7] Gosselin R (2011). Effect of telavancin (Vibativ) on routine coagulation test results. Am. J. Clin. Pathol..

[8] Belley A (2017). Effects of oritavancin on coagulation tests in the clinical laboratory. Antimicrob. Agents Chemother..

[9] Gosselin RC (2014). Effects of pentasaccharide (fondaparinux) and direct thrombin inhibitors on coagulation testing. Arch. Pathol. Lab. Med..

[10] de Oliveira LM (2010). Fructose-1,6-bisphosphate inhibits in vitro and ex vivo platelet aggregation induced by ADP and ameliorates coagulation alterations in experimental sepsis in rats. J. Thromb. Thromb..

[11] Cavallini L, Deana R, Francesconi MA, Alexandre A (1992). Fructose-1,6-diphosphate inhibits platelet activation. Biochem. Pharmacol..

[12] Ruzzene M, Donella-Deana A, Alexandre A, Francesconi MA, Deana R (1991). The antioxidant butvlated hydroxytoluene stimulates’ platelet protein kin&e C and inhibits subsequent protein phosphorylation induced by thrombin. Biochim. Biophys. Acta.

[13] Doni MG, Alexandre A, Padoin E, Bertoncello S, Deana R (1991). ‘Nitrovasodilarors and cGMP inhibit human platelet activation. Cardioscience.

[14] Barriere SL (2011). Effects of telavancin on coagulation test results. Int. J. Clin. Pract..

[15] Farias LA, Smith EE, Markov AK (1990). Prevention of ischemic-hypoxic brain injury and death in rabbits with fructose-1,6-diphosphate. Stroke.

[16] Platton S (2018). A multicenter study to evaluate automated platelet aggregometry on Sysmex CS-series coagulation analyzers-preliminary findings. Res. Pract. Thromb. Haemost..

